# HIV-related stigma and discrimination amongst healthcare providers in Guangzhou, China

**DOI:** 10.1186/s12889-018-5654-8

**Published:** 2018-06-15

**Authors:** Xiaomei Dong, Jianwei Yang, Lin Peng, Minhui Pang, Jiayi Zhang, Zhan Zhang, Jiaming Rao, Haiqing Wang, Xiongfei Chen

**Affiliations:** 1 Department of Epidemiology, School of Basic Medicine, Jinan University, Guangzhou, China; 2 Dongguan Municipal Centers for Disease Control and Prevention, Dongguan, Guangdong Province China; 3 Department of Medical Statistics, School of Basic Medicine, Jinan University, Guangzhou, China; 4 Guangzhou Centers for Disease Control and Prevention, Guangzhou, Guangdong China

**Keywords:** HIV/AIDS, Healthcare providers, Stigma and discrimination

## Abstract

**Background:**

HIV-related discrimination amongst healthcare providers is one of the strongest obstacles to effectively responding to HIV. This study was conducted to explore the occurrence of and other factors related to discrimination against people living with HIV/AIDS amongst healthcare providers in Guangzhou, China.

**Methods:**

This was a cross-sectional study, conducted between July and October 2016, that enrolled healthcare providers from 9 healthcare institutions in Guangzhou, China. HIV-related discrimination was assessed using anonymous self-designed questionnaires. Chi-square tests were used to study the differences in the socio-demographic characteristics, occupational characteristics, HIV-related knowledge and personal attitudes between participants who had and had not discriminated against People living with HIV/AIDS (PLWHA). A multivariate logistic regression analysis was used to study the factors associated with HIV-related discrimination.

**Results:**

A total of 972 healthcare providers were investigated, and 386 (39.7%) had previously served HIV-positive individuals in their work. Administering HIV antibody tests for patients without his or her consent was the most frequent act of discrimination (65.3%), and other forms of discrimination, including “differential treatment” (51.0%), “disclosed information” (46.4%) and “refused to treat” (38.6%), were also prevalent. The logistic regression analysis indicated that people who had worked for 3–7 years, worked in secondary hospitals or lower, worked in surgical departments, had lower scores on HIV transmission knowledge, were dissatisfied with the occupational exposure protection system offered by the government, were worried about HIV-related exposure and feared HIV-related exposure were more likely to commit an act of medical discrimination against PLWHA.

**Conclusion:**

HIV-related discrimination was not unusual in the healthcare providers of Guangzhou, which may be related to their negative cognitions and attitudes as well as the hospital management system and government policy. Therefore, comprehensive HIV-related knowledge education should be implemented to change the attitude of healthcare providers. In addition, the current laws and regulations should be refined by the government to protect the rights of healthcare providers. The contradiction between designated hospitals and non-designated hospitals should be resolved to ensure that PLWHA receive timely and effective help and treatment.

## Background

HIV stigma is prevalent worldwide. The Joint United Nations Program on HIV/AIDS (UNAIDS) defines HIV-related stigma and discrimination as follows: “a process of devaluation of people either living with or associated with HIV and AIDS” [1, 2]. HIV/AIDS-related stigma and discrimination occur in many settings, but they may have more serious consequences in healthcare settings [3–5]. In recent years, many media outlets have frequently portrayed HIV-positive patients encountering several difficulties in the process of seeking medical treatment in China, particularly the case of Xiao Feng, an AIDS patient. To receive a surgical procedure that two hospitals denied him, he eventually received the surgery he needed after concealing his HIV-positive status, which propelled public discussion [6–8].

“HIV is dreadful, but discrimination is even more dreadful,” said Cai Weiping, a director of the infectious diseases department at Guangzhou No. 8 People’s Hospital. Stigmatizing attitudes and behaviours towards people living with HIV/AIDS (PLWHA) not only interfere with their decisions to seek HIV counselling and testing, the prevention of mother-to-child transmission, and the likelihood of disclosing their HIV status [3, 9], but these attitudes and behaviours can also hinder the progress of AIDS prevention and control. In addition, discrimination from the outside world will lead to negative emotions amongst PLWHA, including anxiety, depression, guilt, and other mental health symptoms [10, 11].

In China, to ensure that AIDS patients have access to medical care, employment and privacy, the government has developed a number of laws and regulations, such as the Law of the People’s Republic of China on the Prevention and Treatment of Infection Diseases (2004), the Regulations on AIDS Prevention and Treatment (2006) [12], and the Tort Law of the People’s Republic of China (2009) [13]. However, the government’s guide on how hospitals should receive HIV/AIDS patients is not sufficiently clear, and some medical facilities use the designated hospitals as an excuse to reject patients from receiving routine medical services. The designated hospitals are selected by the Ministry of Health to treat PLWHA, particularly those requiring antiviral treatment. However, most designated hospitals are specialized hospitals that only address infectious diseases and are not qualified to perform major surgeries. This phenomenon may lead to concealed illnesses of patients and cause irreparable damage to society. Therefore, the serious problem of HIV/AIDS discrimination commands attention [14, 15].

HIV stigma and discrimination have been extensively documented amongst healthcare workers (HCWs) worldwide [5, 16–19]. To combat stigma and discrimination, it is important to explore their associated factors and to explore how they vary across cultural contexts within a country [20]. Nevertheless, in the Chinese context, HIV-related discrimination in medical institutions was conducted mainly amongst PLWHA, and fewer studies have investigated the phenomena from the perspective of HCWs. Moreover, most studies focus on discriminatory attitudes, with occasional studies involving specific discriminatory behaviours. In this study, we selected medical staff members as participants, investigated their attitudes and behaviours towards HIV-positive patients, and explored the influencing factors of discriminatory behaviours, which will provide countermeasures and references for relevant departments to reduce discrimination in the medical field, protect the medical interests of PLWHA and promote HIV prevention and control.

## Methods

### Study population, setting and design

This study is a cross-sectional survey of a convenience sample of 972 health care providers from different grades of healthcare facilities in Guangzhou. The study was conducted in 2016 with the goal of assessing HIV-related discrimination and behavioural risk factors in healthcare providers. Guangzhou is located in the Pearl River Delta area and has a high HIV infection rate in China. To meet the inclusion criteria, the participants were required to have worked as a doctor, nurse or laboratory staff member in a current healthcare setting for at least one year and be able to answer questions without assistance. All participants provided written informed consent. All procedures were approved by the Ethical Committee of the Guangzhou Centers for Disease Control and Prevention.

### Sampling procedure

The sample content was calculated according to the following formula: n = z^2^ p (1-p)/d^2^. Our study established a 95% confidence level (z = 1.96); tolerance was defined as 0.03. According to the pre-investigation results, the incidence of medical discrimination was 70% (*P* = 0.7, 1-*P* = 0.3). To reduce the problem of an insufficient sample size caused by missing or incomplete data from the questionnaire, the sample size was increased by 20% from the original. In total, the sample size was 1075. Considering the classified management system of medical organizations issued by the Ministry of Health of the People’s Republic of China in 1989, public hospitals are classified into three grades (tertiary hospitals are better than secondary hospitals, which are in turn better than primary hospitals). To improve the sample representation, a stratified sampling method was adopted. We selected 4 tertiary hospitals, 3 secondary hospitals and 2 primary hospitals. Taking into account the personnel size and department settings in the different levels of hospitals, 150–200 questionnaires were distributed in the tertiary hospitals, 80–100 questionnaires were distributed in the secondary hospitals, and 30–50 questionnaires were distributed in the primary hospitals. A total of 1075 questionnaires were distributed; 1031 questionnaires were withdrawn, and 972 completed questionnaires were obtained, yielding response and completion rates of 95.91 and 94.28%, respectively.

### Data collection procedure

The data concerning the HCWs’ attitudes and acts towards PLWHA were collected using a self-administered questionnaire, which was created on the basis of literature references, pilot tests and expert optimization [3–5, 21]. The investigation was conducted with the help of the Department of Hospital Infection Management in each hospital. The questionnaires were completed by the medical staff during leisure time at work. Before the formal investigation, the 972 HCWs who were eligible for enrolment were asked to sign the informed consent form. The questionnaire took an average of 15 min to complete. After the HCWs independently finished their own questionnaire, we reviewed the questionnaire and then arranged and numbered the questionnaires at that time.

### Measures

The questionnaires comprised 4 parts, including socio-demographic variables, occupational characteristics, HIV-related knowledge, personal attitudes and discrimination behaviours.

#### HIV-related knowledge

HIV-related knowledge contained 2 parts, including HIV transmission knowledge and exposure prevention knowledge (items are listed in Table 2).

- The HIV transmission knowledge section included 6 questions. For each item, the responses were coded as 1 (correct answer) or 0 (incorrect answer or unknown). The number of correct responses was summed. A higher score reflected better knowledge. Because the median knowledge score was 4, the respondents with correct answers to at least 4 questions represented the high-score group, and those with correct answers to less than 4 questions represented the low-score group.
- Exposure prevention knowledge was used to measure the emergency response ability of the medical staff after HIV exposure at work. This module contained 10 questions. Respondents with correct responses to at least six of the ten exposure prevention knowledge questions were defined as the high-score group, and those with less than 6 correct responses were defined as the low-score group.

#### Personal attitudes

The personal attitudes section contained 3 parts, including willingness to treat, satisfaction with the security system and fear of HIV exposure.

- Willingness to treat: Two individual items measured the medical workers’ willingness to take care of PLWHA: (i) general treatment and (ii) high-risk treatment (yes or no).
- Evaluation of occupational exposure protection system: The evaluation of the current security system offered by the government for HIV occupational exposure had one question measured on a three-point scale (1 = not at all to 3 = very satisfied).
- Fear of HIV exposure: The fear of work-related HIV exposure had one question measured on a three-point scale (1 = no fear, 2 = some fear, and 3 = substantial fear).

#### Discrimination behaviours

This module was completed by participants who had experience in the diagnosis and treatment of PLWHA. Guided by the Protocol for the Identification of Discrimination against People Living with HIV (UNAIDS, 2000), the following four questions about HIV-related discrimination in health care were included: (1) Did you ever refuse to treat patients on the grounds of HIV/AIDS status (refused to treat)? (2) Did you ever administer an HIV antibody test for patients without his or her consent (forced detection)? (3) Did you ever administer a different treatment for patients on the grounds of HIV/AIDS status (differential treatment)? (4) Did you ever disclose a patient’s HIV status to a colleague who was not directly involved in the management of that case (disclosed information)? The response options for each statement were yes or no.

### Statistical analysis

The data were checked for integrity and were entered into Epi-data 3.1. The data analysis was performed using SPSS 19.0. Descriptive statistics were used to describe the health care providers’ demographic characteristics and occupational characteristics. In addition, we explored differences in the socio-demographic characteristics, occupational characteristics, HIV-related knowledge and personal attitudes between participants who had and had not discriminated against PLWHA by using chi-square tests (α=0.05). Furthermore, a multivariate logistic regression analysis was used to study the related factors of four types of HIV-related discrimination, and the factors were screened by the likelihood ratio approach method (α=0.05).

## Results

### Sociodemographic and clinical characteristics

As shown in Table 1, 972 healthcare workers were included in the sample. There were 706 females, representing 72.6% of the sample. Approximately 44.2% of the respondents had been working at their current health care setting for less than 2 years, 31.6% had been working there for more than 8 years, 66.4% of the sample came from tertiary hospitals, and 45.0% of the participants were doctors. Amongst all participants, 53.4% reported having received training on occupational exposure protection skills, and 59.2% had received an undergraduate education or above.

**Table 1:** Sociodemographic characteristics of healthcare providers, Guangzhou, China

Variable	Number	Percent
Gender		
Male	266	27.4
Female	706	72.6
Working years		
≤2 years	430	44.2
3–7 years	235	24.2
≥8 years	307	31.6
Medical education		
Junior college degree or lower	396	40.7
Medical degree	428	44.0
Higher than medical degree	148	15.2
Level of care		
Tertiary hospital	645	66.4
Secondary hospital or lower	327	33.6
Training		
Yes	519	53.4
No	453	46.6
Profession		
Doctor	437	45.0
Nurse	432	44.4
Laboratory technician	103	10.6
Department		
Internal medicine	266	27.4
Surgical department	355	36.5
Clinical laboratory department	66	6.8
Administrative department	285	29.3

### HIV-related knowledge

The aggregate knowledge score was 10.74 (67.13 out of 100). The mean HIV transmission knowledge score was 4.09. Knowledge regarding the route of exposure was high, with 84.2% of respondents answering accurately that the disease could not be spread by intact skin coming in contact with HIV-infected blood. Knowledge was somewhat deficient regarding the AIDS epidemic in China. The results for HIV transmission knowledge by item are displayed in Table 2. The mean score of HIV exposure knowledge was 6.65. The item with the highest rate of correct answers was “How should one address needles used by HIV-positive patients?” The item with the lowest rate of correct answers was “Which is the correct operation to prevent occupational exposure to HIV?” The results for exposure prevention knowledge by item are displayed in Table 2.

**Table 2:** HIV-related knowledge of healthcare providers, Guangzhou, China

	Item	Correct responses N (%)
HIV transmission knowledge	Which is the least risky pattern of exposure to HIV-positive blood?	818 (84.2)
	Which type of body fluid from PLWHA can cause infection?	797 (82.0)
	Which is the most effective measure to prevent HIV infection?	769 (79.1)
	What is the current stage of the AIDS epidemic in China?	752 (77.4)
	Which sexual behaviour is the most dangerous?	470 (48.4)
	What are the epidemic characteristics of HIV/AIDS in China?	379 (39.0)
Exposure prevention knowledge	How should one address needles used by HIV-positive patients?	879 (90.4)
	Which is not a universal precautionary principle to prevent occupational exposure to HIV?	807 (83.0)
	Which is not a measure of HIV occupational exposure?	741 (76.2)
	What is the best time for preventive medication after occupational exposure?	720 (74.1)
	Which treatment is not correct if stabbed by HIV-contaminated needles?	681 (70.1)
	Which is not a principle that is commonly followed by parties and the relevant departments after HIV occupational exposure?	564 (58.0)
	Which is not the main basis for assessing the risk of HIV exposure?	556 (57.2)
	How many days is the course of preventive medication after occupational exposure?	491 (50.5)
	How should one to treat the eye mucosa after it is splashed by HIV-infected fluid?	460 (47.3)
	Which is the correct operation to prevent occupational exposure to HIV?	214 (22.0)

### Personal attitudes at work

The participants’ attitudes towards PLWHA are presented in Table 3. In total, 33.0% of healthcare workers reported that they were satisfied with the protection system offered by the government for HIV occupational exposure, whereas 57.2% were neutral regarding the system. Approximately 80% of participants indicated that they were worried about work-related HIV transmission. When asked about the willingness to treat HIV-positive patients, 85.2% of participants responded that they would provide general treatment for PLWHA, whereas 78.7% would provide high-risk treatment for PLWHA.

**Table 3:** Healthcare providers’ personal attitudes at work

Personal attitudes	N (%)
Evaluation of occupational exposure protection system	
Satisfaction	321 (33.0%)
Neutral	557 (57.2%)
Dissatisfaction	94 (9.7%)
Fear of work-related HIV exposure	
Substantial fear	158 (16.3%)
Some fear	611 (62.9%)
No fear	203 (20.9%)
I would provide general treatment for PLWHA	
Yes	828 (85.2%)
No	144 (14.8%)
I would provide high-risk treatment for PLWHA	
Yes	765 (78.7%)
No	207 (21.3%)

### Discrimination against PLWHA

Amongst the 972 respondents, 386 (39.7%) reported they had experience in the diagnosis and treatment of PLWHA, whereas 586 (60.3%) had no such experience. We found that 300 (77.72%) HCWs had discriminated against PLWHA in the process of providing medical attention. Administering HIV antibody tests for patients without his or her consent was the act of discrimination reported by the greatest proportion of healthcare providers (65.3%). The act of discrimination that was reported by the second-highest proportion of healthcare providers (51.0%) was providing differential treatment to patients based on HIV status. Furthermore, 46.4% of healthcare providers indicated that they had disclosed a patient’s HIV status to a colleague who had not been directly involved in the management of the case, and 38.6% reported that they had refused to treat PLWHA in the past (Table 4).

**Table 4:** Acts of discrimination by healthcare providers, Guangzhou, China

	Had experience in the diagnosis and treatment of PLWHA			Had no experience
	Yes	No	Total	
Refused to treat	149 (38.6)	237 (61.4)	386	586
Forced detection	252 (65.3)	134 (34.7)	386	586
Differential treatment	197 (51.0)	189 (49.0)	386	586
Disclosed information	179 (46.4)	207 (53.6)	386	586

### Bivariate results

Table 5 shows the results of the bivariate associations between discrimination acts and factors. Regarding clinic-level factors, the healthcare providers who worked in tertiary hospitals refused to treat PLWHA and conducted HIV tests without the patients’ consent less frequently than those who worked in secondary hospitals or lower (*P* < 0.05). Work seniority differences were found only in the “refused to treat” category, with the workers with 3–7 years of experience being more likely to refuse to treat PLWHA (*P* < 0.05). Healthcare providers who worked in surgical departments reported higher rates of “forced detection” (*P* < 0.01) and “differential treatment” (*P* < 0.01) than those of participants in internal medicine departments.

**Table 5:** Univariate analysis of discrimination towards PLWHA, Guangzhou, China

	Refused treatment (*n* = 386)			Forced detection (n = 386)			Differential treatment (n = 386)			Disclosed information (*n* = 386)		
	Yes	No	*χ* ^*2*^	Yes	No	*χ* ^*2*^	Yes	No	*χ* ^*2*^	Yes	No	*χ* ^*2*^
Level of care												
Tertiary hospital	87 (31.3)	191 (68.7)	22.38a	171 (61.5)	107 (38.5)	6.23 b	142 (51.1)	136 (48.9)	0.00	130 (46.8)	148 (53.2)	0.06
Secondary hospital and lower	62 (57.4)	46 (42.6)		81 (75.0)	27 (25.0)		55 (50.9)	53 (49.1)		49 (45.4)	59 (54.6)	
Number of years working												
≤ 2 years	46 (34.6)	87 (65.4)	6.84 b	85 (63.9)	48 (36.1)	0.19	68 (51.1)	65 (48.9)	0.03	68 (51.1)	65 (48.9)	3.19
3–7 years	55 (48.7)	58 (51.3)		74 (65.5)	39 (34.5)		57 (50.4)	56 (49.6)		45 (39.8)	68 (60.2)	
≥ 8 years	48 (34.3)	92 (65.7)		93 (66.4)	47 (33.6)		72 (51.4)	68 (48.6)		66 (47.1)	74 (52.9)	
Profession												
Doctor	63 (37.3)	106 (62.7)	3.26	105 (62.1)	64 (37.9)	5.13	91 (53.8)	78 (46.2)	2.15	77 (45.6)	92 (54.4)	0.35
Nurse	82 (41.4)	116 (58.6)		138 (69.7)	60 (30.3)		99 (50.0)	99 (50.0)		92 (46.5)	106 (53.5)	
Laboratory technician	4 (21.1)	15 (78.9)		9 (47.4)	10 (52.6)		7 (36.8)	12 (63.2)		10 (52.6)	9 (47.4)	
Department												
Internal medicine	48 (37.2)	81 (62.8)	6.14	71 (55.0)	58 (45.0)	11.93^a^	52 (40.3)	77 (59.7)	11.27^a^	66 (51.2)	63 (48.8)	3.88
Surgical department	65 (38.9)	102 (61.1)		119 (71.3)	48 (28.7)		100 (59.9)	67 (40.1)		68 (40.7)	99 (59.3)	
Clinical laboratory department	3 (15.8)	16 (84.2)		10 (52.6)	9 (47.4)		9 (47.4)	10 (52.6)		10 (52.6)	9 (47.4)	
Administrative department	33 (46.5)	38 (53.5)		52 (73.2)	19 (26.8)		36 (50.7)	35 (49.3)		35 (49.3)	36 (50.7)	
HIV transmission knowledge												
Low score	96 (43.8)	123 (56.2)	5.85^b^	157 (71.7)	62 (28.3)	9.16^b^	115 (52.5)	104 (47.5)	0.44	113 (51.6)	106 (48.4)	5.56 b
High score	53 (31.7)	114 (68.3)		95 (56.9)	72 (43.1)		82 (49.1)	85 (50.9)		66 (39.5)	101 (60.5)	
Exposure prevention knowledge												
Low score	97 (42.9)	129 (57.1)	4.29^b^	152 (67.3)	74 (32.7)	0.94	122 (54.0)	104 (46.0)	1.89	110 (48.7)	116 (51.3)	1.16
High score	52 (32.5)	108 (67.5)		100 (62.5)	60 (37.5)		75 (46.9)	85 (53.1)		69 (43.1)	91 (56.9)	
Training												
No	74 (44.3)	93 (55.7)	4.05^b^	111 (66.5)	56 (33.5)	0.18	92 (55.1)	75 (44.9)	1.94	80 (47.9)	87 (52.1)	0.28
Yes	75 (34.2)	144 (65.8)		141 (64.4)	78 (35.6)		105 (47.9)	114 (52.1)		99 (45.2)	120 (54.8)	
Evaluation of occupational exposure protection system												
Satisfaction	62 (44.0)	79 (56.0)	2.82	89 (63.1)	52 (36.9)	8.27 a	64 (45.4)	77 (54.6)	2.85	63 (44.7)	78 (55.3)	0.44
Neutral	70 (35.0)	130 (65.0)		125 (62.5)	75 (37.5)		109 (54.5)	91 (45.5)		96 (48.0)	104 (52.0)	
Dissatisfaction	17 (37.8)	28 (62.2)		38 (84.4)	7 (15.6)		24 (53.3)	21 (46.7)		20 (44.4)	25 (55.6)	
Fear												
Substantial fear	41 (54.7)	34 (45.6)	10.16 a	67 (89.3)	8 (10.7)	27.43 a	58 (77.3)	17 (22.7)	26.12 a	43 (57.3)	32 (42.7)	4.52
Some fear	80 (34.5)	152 (65.5)		131 (56.5)	101 (43.5)		106 (45.7)	126 (54.3)		102 (44.0)	130 (56.0)	
No fear	28 (35.4)	51 (64.6)		54 (68.4)	25 (31.6)		33 (41.8)	46 (58.2)		34 (43.0)	45 (57.0)	

^b^ *P* < 0.05, ^a^ *P* < 0.01

Concerning the level of HIV-related knowledge, the participants who had higher scores on HIV transmission knowledge reported higher rates of “refused treatment” (*P* < 0.05), “forced detection” and “disclosed information” (*P* < 0.05). In addition, those who reported higher scores on exposure prevention knowledge had higher rates of “refused treatment” (*P* < 0.05). With regard to personal attitudes, the people who were more satisfied with the current occupational exposure protection system had a smaller possibility of having conducted HIV tests without consent (*P* < 0.01). In addition, fearing work-related HIV exposure was a significant predictor of “refused treatment” (*P* < 0.01), “forced detection” (*P* < 0.01) and “differential treatment” (*P* < 0.01).

### Multivariate results

The multivariate regression results are displayed in Table 6. Four models included variables that were significant in their univariate analysis. In the multivariate model of “refused treatment”, all factors that were significant in the univariate analysis, in addition to exposure prevention knowledge and training, remained significant influential factors of discrimination. Working in secondary hospitals or lower, working for 3–7 years, having lower scores on HIV transmission knowledge and fearing HIV-related exposure continued to be significant predictors of higher levels of “refused treatment”. The model of “forced detection” showed that working in surgical departments, working in secondary hospitals or lower, being dissatisfied with the occupational exposure protection system offered by the government and being worried about HIV-related exposure were significant factors of higher rates of “forced detection”. The factors of department and fear of HIV-related exposure were entered in the model of “differential treatment”, and HIV transmission knowledge was entered in the model of “disclosed information”.

**Table 6:** Multivariate regression analysis of discrimination towards PLWHA, Guangzhou

Discrimination	Variables	Wald	OR (95% CI)
Refused treatment (0 = No, 1 = Yes)	Level of care (ref: Tertiary hospital)		
	Secondary hospital or lower	19.405	2.997 (1.839, 4.885)
	Number of years working (ref: ≥8 years)	6.097	
	3–7 years	5.179	1.877 (1.091, 3.227)
	≤2 years	0.080	1.083 (0.624, 1.881)
	HIV transmission knowledge (ref: High score)		
	Low score	5.797	1.741 (1.109, 2.735)
	Fear (ref: No fear)	9.316	
	Some fear	0.369	0.840 (0.478, 1.474)
	Substantial fear	4.115	2.021 (1.024, 3.987)
Forced detection (0 = No, 1 = Yes)	Level of care (ref: Tertiary hospital)		
	Secondary hospital or lower	5.444	1.900 (1.108, 3.258)
	Department (ref: Internal medicine)	12.558	
	Surgical department	9.202	2.221 (1.326, 3.720)
	Clinical laboratory department	0.411	0.710 (0.249, 2.024)
	Administrative department	4.249	2.031 (1.035, 3.985)
	Evaluation of Occupational Exposure Protection System (ref: Satisfaction)	6.638	
	Neutral	0.053	1.058 (0.653, 1.714)
	Dissatisfaction	6.320	3.276 (1.299, 8.262)
	Fear (ref: No fear)	23.348	
	Some fear	3.440	0.585 (0.332, 1.031)
	Substantial fear	9.113	4.014 (1.628, 9.898)
Differential treatment (0 = No, 1 = Yes)	Department (ref: Internal medicine)	13.779	
	Surgical department	13.490	2.520 (1.539, 4.127)
	Clinical laboratory department	0.393	1.383 (0.502, 3.815)
	Administrative department	1.647	1.492 (0.810, 2.751)
	Fear (ref: No fear)	25.99	
	Some fear	0.819	1.277 (0.752, 2.169)
	Substantial fear	21.741	5.583 (2.710, 11.504)
Disclosed information (0 = No, 1 = Yes)	HIV transmission knowledge (ref: High score)		
	Low score	5.527	1.631 (1.085, 2.453)

## Discussion

In the medical field, discrimination and stigma have engendered serious and even tragic consequences, denying the people living with HIV/AIDS access to care and testing, which can in turn increase the likelihood of patients concealing their HIV status [8, 22]. In addition, the chronic nature of HIV necessitates ongoing medical care across a lifespan, increasing the chances of a doctor being exposed to the disease; however, the patient will not receive proper treatment if doctors do not know his or her infection status [23]. In addition, stigma and discrimination have been extensively reported in medical settings worldwide, most of which involved discriminatory attitudes amongst medical workers, with a few cases of discriminatory behaviour. Therefore, it is critical to study the relationship between the attitudes and behaviours of individuals who engage in HIV-related discrimination and to explore the causes of this discrimination.

In our study, we found that 77.7% of HCWs had discriminated against PLWHA in the process of providing medical attention. One of the most commonly reported forms of discrimination was forced detection, followed by differential treatment, disclosed information and refused treatment. All rates were higher than those reported in a Belizean study by Andrewin A [24]. The reason for these higher rates may be related to the contradiction between designated hospitals and non-designated hospitals that was mentioned previously because doctors always transfer PLWHA to designated hospitals. Additionally, it was noted that healthcare providers give differential treatment and disclose HIV status to facilitate the healthcare given to the clients and to take protective measures when dealing with PLWHA [5, 25]. However, these acts could be considered discrimination and privacy violations by PLWHA, resulting in irreparable psychological damage to those patients. Therefore, we must determine how to reduce unintentional discrimination in future studies.

Occupational characteristics were found to be significantly associated with acts of HIV-related discrimination in this population. Participants in secondary hospitals or lower had a higher frequency of “refused treatment”. In China, compared to tertiary hospitals, which have more advanced equipment and more highly educated staff members, primary hospitals are limited by materials and supplies, and they cannot provide simple treatments, such as haemorrhoid surgery [26]. Similarly, the healthcare providers in surgical departments had a higher frequency of “forced detection” and “differential treatment”. The Twelfth Five-Year Action Plan for HIV/AIDS Prevention and Control in China pointed out that routine examination for inpatients and outpatients will include HIV testing in highly endemic HIV areas. Because it is routine testing before surgery, medical staff members often forget to inform the patient, thus violating the HIV-positive patient’s rights. Therefore, we strongly recommend that providers increase interactions with PLWHA before treatment.

Negative attitudes of health professionals, which are key factors in dealing with PLWHA, may result in undesirable consequences [27]. In the present study, the people who were more satisfied with the occupational exposure protection system had a lower frequency of “forced detection”. One study amongst healthcare providers in Southwest Ethiopia, for example, found that the perception of protocol-related institutional support could reduce the stigma score ^5^. Another study by LiLi detected similar trends [28]. These findings highlight the importance of establishing institutional support in the work of eliminating discrimination. However, only one-third of respondents in this survey were satisfied with the occupational exposure protection system. Thus, most hospitals lack a strict series of AIDS occupational protection training, protective equipment or rules and regulations, which objectively hinders the supply of medical personnel health services.

In our study, fear of occupational exposure was a key factor in the occurrence of medical discrimination. Other studies have also shown that worry about infection at work favours discriminatory attitudes ^3^. Therefore, reducing fear is also an urgent problem to be addressed. To maintain the interests of the medical staff members and eliminate their fears, the National Health and Family Planning Commission of the PRC released the latest version of the “Occupational Diseases Classification and Catalog” with the inclusion of AIDS (limited to health staff and policemen) for the first time in 2013; however, the amount of compensation is not clear, and the process is complicated. Therefore, the claims system must be further improved. In addition, preventive measures should also be strengthened. From the aspects of medical staff members, HIV-related training should be conducted to address unwarranted fears about the risk of infection with HIV after casual contact. From the governmental perspective, the medical investment should be increased to ensure that there are adequate hardware facilities to prevent the infection of healthcare workers.

Similar to other studies, ours has several limitations that must be considered when interpreting the results. The data were collected from only one city; thus, caution should be exercised in generalizing these findings to a different population or other geographic locations. However, Guangzhou, as the capital city of Guangdong Provence, has a wide range of services. In addition, we made every effort to recruit healthcare providers from different levels of hospitals. Therefore, our findings provide important support for the need for HIV-related stigma-reduction interventions in the Chinese healthcare setting.

## Conclusions

Our findings revealed that HIV-related discrimination was not unusual in the healthcare providers of Guangzhou, including “Refused to treat”, “Forced detection”, “Differential treatment”, and “Disclosed information”, which were related to the health care workers’ negative cognitions and attitudes, the hospital management system and government policy. Therefore, future treatment and support for AIDS patients should focus more on changing the HCWs’ cognitions and attitudes towards PLWHA to ensure that PLWHA can receive timely and effective help and treatment. The reduction of medical discrimination was inseparable from government policy support and the hospital management system. The government should implement measures to refine the current laws and regulations and establish a professional insurance mechanism to protect the rights of medical staff members, thereby reducing the worries of HCWs in providing services to AIDS patients. The hospital should attend to the contradiction between designated hospitals and non-designated hospitals, increase investment and improve service capacity to address the rejection caused by the lack of technology and equipment.

## Abbreviations

HCW: Healthcare worker
PLWHA: People living with HIV/AIDS
PRC: People’s Republic of China
UNAIDS: The Joint United Nations Program on HIV/AIDS
